# Sensitivity of human auditory cortex to rapid frequency modulation revealed by multivariate representational similarity analysis

**DOI:** 10.3389/fnins.2014.00306

**Published:** 2014-09-30

**Authors:** Marc F. Joanisse, Diedre D. DeSouza

**Affiliations:** Department of Psychology, Brain and Mind Institute, The University of Western OntarioLondon, ON, Canada

**Keywords:** frequency modulation, auditory cortex, heschl's gyrus, multivoxel pattern analysis (MVPA), functional magnetic resonance imaging (fMRI)

## Abstract

Functional Magnetic Resonance Imaging (fMRI) was used to investigate the extent, magnitude, and pattern of brain activity in response to rapid frequency-modulated sounds. We examined this by manipulating the direction (rise vs. fall) and the rate (fast vs. slow) of the apparent pitch of iterated rippled noise (IRN) bursts. Acoustic parameters were selected to capture features used in phoneme contrasts, however the stimuli themselves were not perceived as speech *per se*. Participants were scanned as they passively listened to sounds in an event-related paradigm. Univariate analyses revealed a greater level and extent of activation in bilateral auditory cortex in response to frequency-modulated sweeps compared to steady-state sounds. This effect was stronger in the left hemisphere. However, no regions showed selectivity for either rate or direction of frequency modulation. In contrast, multivoxel pattern analysis (MVPA) revealed feature-specific encoding for direction of modulation in auditory cortex bilaterally. Moreover, this effect was strongest when analyses were restricted to anatomical regions lying outside Heschl's gyrus. We found no support for feature-specific encoding of frequency modulation rate. Differential findings of modulation rate and direction of modulation are discussed with respect to their relevance to phonetic discrimination.

## Introduction

During verbal communication, our auditory system is charged with the task of sorting through a complex acoustic stream in order to identify relevant stimulus features, and then integrating this information into a unified phonetic percept that can allow us to perceive the incoming message. This process occurs amidst competing sources of information and significant variability in how a given speech sound is produced both within- and between-speakers. Yet humans can decode auditory speech both accurately and in a way that usually seems effortless.

A key characteristic of the speech signal is that it contains acoustic complexities in both the spectral and temporal domains. Spectrally, it contains simultaneous bands of high and low intensities across a range of frequencies. Temporally, the signal is amplitude modulated such that its intensity is rapidly changing and fast fading, and it is frequency modulated so that spectral information changes at a rapid rate. This multicomponent nature of the acoustic speech signal makes it unique in the domain of auditory processing.

In the present study we focus on the neural processing of one specific characteristic of temporal-acoustic speech processing, namely rapid frequency modulation (FM). The production of many phonemes results in a concentration of resonant frequencies, known as formants. The frequencies of these formants will vary depending on the configuration of the vocal tract during articulation. Because speech is produced in a dynamic fashion, formant frequencies tend to rapidly change at differing rates over time (Hillenbrand et al., 1995). Accordingly, manipulating the FM characteristics of formants in speech changes its perceived phonemic characteristics (e.g., Stevens and Klatt, 1974). For example, slowing the rate of a syllable-initial stop consonant's formant transitions will change the perception of /b/ to /w/ (Liberman et al., 1956). Likewise, changing the direction of the second formant's (F2) transition will change a /b/ to /d/ (Miller and Liberman, 1979). Given the important role of formant transitions in speech perception, the present research focuses on examining the neural underpinnings of how these FM acoustic cues are perceived.

Prior work supports the view that auditory cortex in superior temporal gyrus (STG) is organized in a hierarchical fashion that supports the processing of increasingly complex characteristics of auditory signals. Thus, as we move outward from the core region of auditory cortex formed by Heschl's gyrus toward the “belt” and “parabelt” regions that surround it, we observe regions that respond to the increasingly complex spectral and temporal characteristics of acoustic stimuli. Converging support for this notion has come from studies of auditory cortex in humans (Wessinger et al., 2001; Chevillet et al., 2011) and non-humans (Rauschecker et al., 1995; Kaas et al., 1999; Kikuchi et al., 2010), and across a variety of imaging modalities including functional magnetic resonance imaging (fMRI), magnetoencephalography (MEG) and electrophysiology (Mendelson et al., 1993; Tian and Rauschecker, 2004; Godey et al., 2005; Heinemann et al., 2010; Carrasco and Lomber, 2011).

Studies of the sort do seem to have some implications for how the acoustic form of speech is processed. For instance, Chevillet et al. (2011) compared neural responses to sounds of increasing spectral complexity, namely pure tones, broadband noise, and vowel sounds. They found that pure tones elicited activation in Heschl's gyrus, whereas broadband noise elicited activation in both auditory core as well as belt areas both medial and lateral to the auditory core. Lastly, vowel sounds elicited activation in core, belt, and parabelt regions that surround them. This indicates both a greater sensitivity to spectrally complex sounds in primary auditory cortex, and the increasing recruitment of surrounding brain areas as this complexity increases. Note that although the literature generally supports the notion of a hierarchy from core to belt in auditory cortex, there is some suggestion that primary auditory cortex does itself contain regions sensitive to higher-order auditory scenes (Nelken et al., 2003). Thus, one cannot discount the possibility that this region can decode auditory events as complex objects for subsequent recognition.

### Frequency modulation in human auditory cortex

Most of the studies described above have focused on the effect of modulating the spectral complexity of sounds in order to describe the function of primary vs. secondary auditory cortex. Consequently, much less is known about the organization of auditory cortex with respect to rapid temporal FM cues that are also important for speech. Most of what we know about the coding of FM features in auditory cortex comes from single- and multi-unit electrode recordings of auditory cortex in non-humans. These studies have identified evidence of neuronal selectivity to FM vs. acoustically similar steady-state sounds, across several animal species (Mendelson et al., 1993; Nelken and Versnel, 2000; Liang et al., 2002; Washington and Kanwal, 2008; Kusmierek and Rauschecker, 2009). Moreover, neurons may be individually tuned to specific characteristics of these FM sounds. For instance, Mendelson et al. identified neurons in the primary auditory cortex of cats that are systematically distributed according to either the rate and direction of frequency modulation sweeps. Such findings raise the possibility that auditory cortex in humans is also organized in a way that is preferentially sensitive to these aspects of FM sounds.

Neuroimaging studies in humans have also identified regions of auditory cortex that show a preference to time-varying sounds. For instance, Zatorre and Belin (2001) used positron emission tomography (PET) to examine both the spectral and temporal variation of sounds within human auditory cortex by playing sequences of steady-state pure tones of differing frequencies or durations. The authors found bilateral activation of the core auditory cortex as the rate of pitch variation increased, and bilateral activation of anterior STG in response to spectral variation. Additionally, they found that activation in response to the temporal manipulation was left lateralized while responses to the spectral manipulation were right lateralized. Similarly, Hall and colleagues (Hall et al., 2002) found enhanced fMRI response in STG for FM tone complexes compared to acoustically similar static tones. These FM-sensitive regions included Heschl's gyrus in the left hemisphere, and STG regions adjacent to Heschl's gyrus bilaterally.

There is also some reason to believe that auditory cortex sensitivity to frequency modulation is related to speech processing. Joanisse and Gati (2003) used fMRI to examine activation in superior temporal cortex in response to sequences of stop consonants that varied in their rapid FM characteristics, or vowels that varied in terms of steady-state spectral characteristics. A pair of control conditions used sets of pure tones that also differed along either FM or spectral dimensions. They found that consonants and FM tones yielded stronger activation in left STG and surrounding areas, whereas a congruent effect in the right hemisphere was observed for vowel and pure tone pitch discrimination. The findings again suggest some special status for FM processing in auditory cortex, and that this effect is generally left lateralized.

That said, such studies leave open the possibility that humans maintain cortical regions within primary or secondary auditory cortex that are specially tuned to individual FM features of sounds. Recently Hsieh et al. (Hsieh et al., 2012) examined this issue in humans using fMRI. Their study presented tone complexes that varied in the rate and direction of frequency change; stimuli involved either shorter or longer complex tone sweeps that were either rising or falling. Interestingly, they did not identify brain regions that robustly differentiated either of these two dimensions, suggesting that auditory cortex is not topographically organized in a way that differentiates either the rate nor the direction of FM; that is, no region was more sensitive to rising than falling tones, or showed enhanced activation for faster vs. slower rates of modulation. However, the results were different when the authors employed a multivoxel pattern analysis (MVPA) approach, which takes into account the overall pattern of voxel activity for each stimulus type. This analysis identified (in a subset of subjects) unique patterns of activation for both the rate and direction of FM sweeps in primary auditory cortex and surrounding regions of STG. This suggests that FM-selective brain regions do exist in humans, but that they occur on a level of grain that is much smaller than what can be identified using typical univariate fMRI approaches.

That said, it is not clear how this result bears more narrowly on the question of FM cues for phonemic processing. The stimuli in the Hsieh et al. study involved contrasting relatively slow-going rate changes (0.83 and 3.3 octaves per second) that are not on the order of those used in formant transitions that cue phonemic speech contrasts. Instead the contrasts are more similar to those that signal lexical tone contrasts in some languages; indeed, listeners in their study were native speakers of Mandarin, a tonal language. In addition, the way in which the rate variable was manipulated in this earlier study merits some discussion. FM rate can be modified in three possible ways: the rate of change over time, the extent of frequency change, and the duration of the stimulus itself. However, it is not possible to manipulate one of these independently, and thus two of three factors will always be confounded. Thus, in the Hsieh et al. study the length of the stimulus was manipulated to yield fast vs. slow FM sweeps, such that rate was confounded with overall stimulus duration. This raises the possibility that differences in neural responses to rate reflected sensitivity to the duration of the stimulus rather than the rate of modulation itself. To be clear, such confounds are likely a necessary element of FM stimuli, however it does leave open the possibility that different results could occur when the stimulus rate parameter is manipulated differently.

### Motivation for the present study

Our central focus in the present study was to examine the neural processing of rapid FM features in non-speech acoustic stimuli, compared to acoustically similar steady-state sounds. The intention was to examine how the human brain processes and differentiates characteristics of these stimuli and, in particular, whether different subregions of auditory cortex respond preferentially to these specific features. Consistent with prior studies, FM stimuli in general should yield greater activation both in primary auditory cortex and surrounding regions, when compared to steady-state sounds of similar spectral complexity (Rauschecker et al., 1995; Kusmierek and Rauschecker, 2009). The effect should also be stronger in the left hemisphere. Additionally, we adopted a design that examined differences in neural response to specific features of FM, specifically the direction and rate of change in frequency. This allowed us to assess whether subregions of auditory cortex are specifically tuned to basic features of rapid-FM sounds.

**Figure 1:**
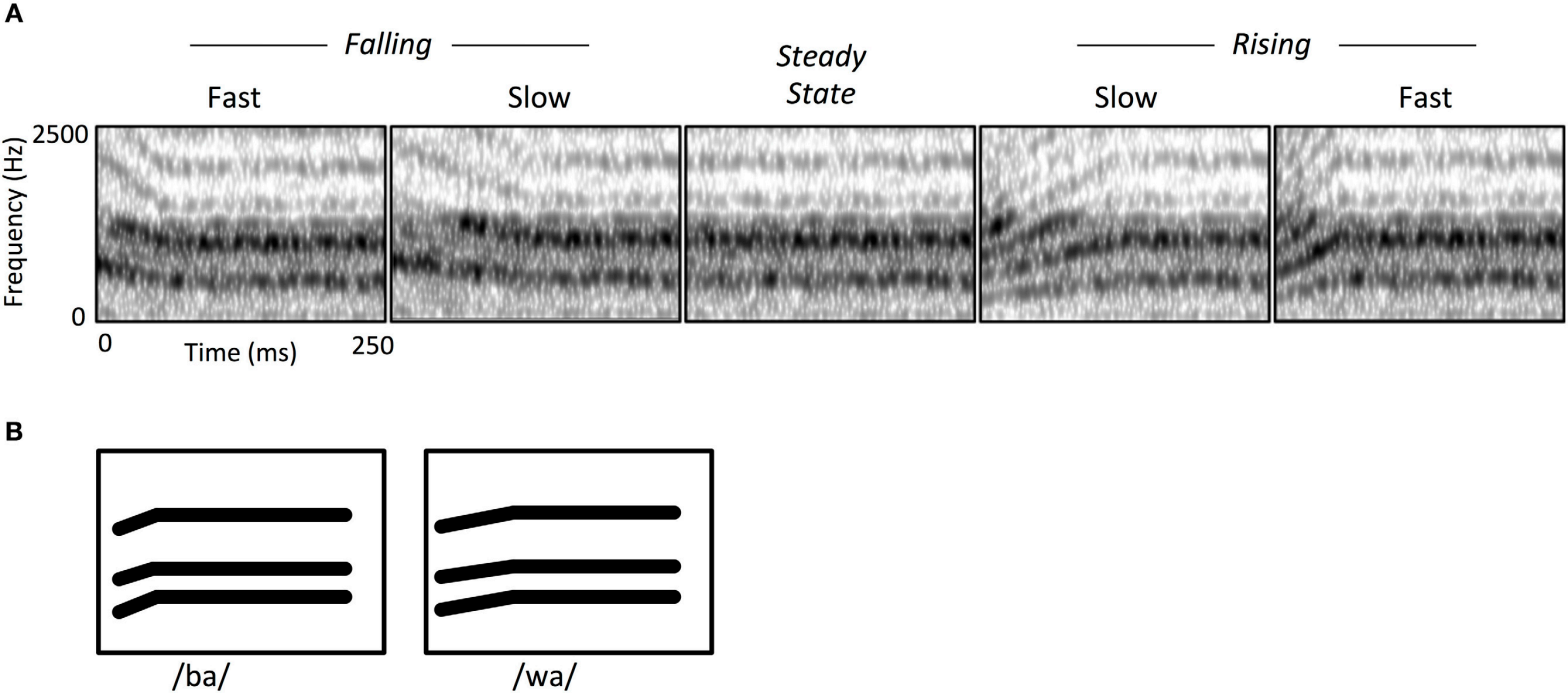
**(A)** Spectrograms of iterative rippled noise (IRN) stimuli, showing frequency modulated (FM) sweeps and steady state conditions. FM stimuli consisted of rising or falling sweeps, at either a fast or short rate of change. All FM stimuli were followed by a steady state portion to hold all stimulus durations at 250 ms. **(B)** Schematized spectrogram of a speech contrast (/ba/ and /wa/) illustrating how the IRN stimuli simulate transition rate contrasts of formant contours in natural speech sounds. Note that while this phoneme pair illustrates a rising contrast, other speech sounds can instead involve falling contrasts.

Central to our approach was using stimuli that capture key acoustic features of speech. Thus, the two FM modulation rates we used roughly correspond to the duration of second-formant transitions observed in stop consonants and semivowels (e.g., /ba/ vs. /wa/; see Figure 1B). We also sought to capture the spectrotemporal complexity of speech by employing iterative rippled noise (IRN) stimuli. IRN is a type of broadband noise that maintains the types of discernible spectral and temporal regularities that are usually associated with narrowband tones (Swaminathan et al., 2008). Just as importantly, IRN does not contain phonetic cues, and does not yield speech-like auditory illusions. This allowed us to capture the general spectral complexity typical of speech, while preserving the ability to manipulate perceived pitch and therefore the FM characteristics of stimuli. IRN stimuli were useful here because they are both spectrally broadband and can contain temporal features mimicking phoneme contrasts, but they are not perceived as speech *per se*. Their spectral characteristics are especially relevant to this end; past research has demonstrated that regions within auditory cortex respond differentially to speech vs. spectrally simple non-speech sounds such as tones (Binder et al., 2000; Whalen et al., 2006). Likewise, IRN stimuli can simulate the high harmonics-to-noise ratio (HNR) of speech (Boersma, 1993). HNR is a higher-order acoustic attribute that indexes the harmonic structure of sounds, and which tends to be higher in natural vocalizations than other types of environmental sounds. There is evidence that subregions of core and belt auditory cortex are specifically tuned to this characteristic due to the increased recruitment of neurons sensitive to multiple frequency-combinations (Lewis et al., 2005). Likewise, there appears to be strong overlap in auditory cortical activity in response to IRN and human vocalizations that is directly attributable to their similarity in HNR (Lewis et al., 2009).

We chose to study FM processing using IRN sounds rather than actual phonetic stimuli in order to avoid potential extraneous influences of speech on the resulting fMRI activation patterns. That is, intelligible speech both comprises acoustic-phonetic information and conveys meaning. Thus, it is challenging to differentiate neural responses to the acoustic features of speech from the effects of its articulatory-phonetic and semantic content. Speech that is intelligible evokes activation in a broader portion of temporal cortex than speech stimuli that have been distorted to the point of unintelligibility (Scott et al., 2000). Likewise, when sinewave tones are systematically combined to approximate the center frequencies of speech formants (i.e., sinewave speech; Remez et al., 1981), listeners can perceive them as having phonetic content. Accordingly, different patterns of activation are observed in temporal cortex when listeners perceive these sinewave sounds as phonetic, compared to when they do not (Liebenthal et al., 2003; Möttönen et al., 2006). Overall, using IRN stimuli allowed us to isolate neural effects of FM processing from effects that occur in response to semantic integration or articulatory-phonetic processing.

We employed two statistical approaches to examine FM processing in auditory cortex. In addition to standard univariate analyses we also used a multivariate approach of representational similarity analysis (RSA). This is an MVPA methodology that computes the similarity of voxel activation patterns among different experimental conditions. While conventional univariate neuroimaging analyses are useful for detecting regional activation differences, they do not provide any information regarding representational differences that occur at a grain of analysis below that afforded by fMRI voxel sizes. On the other hand, MVPA approaches allow us to detect activation patterns in regions of interest even when average activation is similar across conditions (Kriegeskorte et al., 2008). It was therefore expected that RSA could reveal representational differences among FM features in auditory cortex even if univariate analyses failed to reveal large-scale differences in the degree or extent of fMRI activation.

## Methods

### Subjects

Sixteen neurologically healthy adult participants were recruited for this study (eight female, eight male); mean age was 27 years (range 18–31 years). All participants were right-handed, monolingual native English speakers with normal audition by self-report. Informed consent was obtained from each participant in accordance with the University of Western Ontario Medical Research Ethics Board.

### Stimuli

The auditory stimuli consisted of Iterative Rippled Noise (IRN) bursts, which are broadband noise manipulated in a way that produces a perceived pitch contour while maintaining wideband spectral complexity (Figure 1A). Stimuli were created in Matlab (MathWorks, 2010) at a 44.1 KHz sample rate (16-bit quantization), matching the procedure from Swaminathan et al. (2008), whereby a noise impulse is delayed and added to the sample at each iteration, with a delay of 4 ms and a gain of 1. For each stimulus we created a pitch contour represented by a polynomial equation and then created a time varying IRN stimulus that mimicked that input pitch contour by modulating the time delay at each iteration. There were four FM stimulus sweeps in which the center frequency of the IRN was varied linearly over time: Rise-Fast, Rise-Slow, Fall-Fast, and Fall-Slow (Table 1, Figure 1A). The “Fast” sweep had an FM rate of 20 octaves/s and a duration of 50 ms; the FM rate in the “Slow” condition was 10 octaves/s and a 100 ms duration. Note that our goal was to maintain the same duration for all stimuli, which should at least partially overcome the concern that different sweep rates necessarily require either different durations or frequency extents for a stimulus. For that reason, an additional steady-state period was added to the end of each sweep, yielding a total stimulus duration of 250 ms (Figure 1A, Table 1). We also created a fifth “Steady-State” stimulus condition which consisted of an IRN of the same duration and intensity as the FM stimuli, but which had a constant perceived frequency of 1200 Hz.

**Table 1:** **Acoustic characteristics of the IRN stimuli, showing center frequency contours (Hz) for the frequency modulated (FM) and steady-state stimuli**.

	**Time (*ms*)**			
**Condition**	**0**	**50**	**100**	**250**
Rise-Fast	600	1200	1200	1200
Fall-Fast	1800	1200	1200	1200
Rise-Slow	600	900	1200	1200
Fall-Slow	1800	1500	1200	1200
Steady-state	1200	1200	1200	1200

During scanning, stimuli were presented binaurally via MR compatible headphones (Sensimetrics Model S14). Participants were instructed to passively listen to the audio stimuli. A silent movie was displayed via a projector to keep the participant alert. We employed an event-related design in which stimuli were presented at randomly jittered SOAs of 2.1, 4.2, 6.3, or 8.4 s (corresponding to integer multiples of the 2.1 s scan repetition time). A sparse scanning paradigm was used in which silent gaps were introduced between each EPI scan, with auditory stimuli presented during these silent gaps, 50 ms following the end of the previous scan to eliminate possible acoustic masking. The scanning session was divided into six runs with brief rests between each. Within each run, stimuli were presented in pseudo-random order, with 19 presentations of each condition, for a total of 95 presentations per condition over the entire session.

### Neuroimaging

Images were acquired using a 3.0 Tesla Siemens TIM Trio Scanner equipped with a 32-channel head coil. Functional images were acquired in an axial orientation using an iPAT parallel acquisition sequence (GRAPPA, generalized auto-calibrating partially parallel acquisition; acceleration factor 2). Six runs of 252 T2^*^-weighted functional scans were acquired for each subject (voxel size = 3 × 3 × 3 mm; FOV = 192 × 192 mm; *TA* = 1.6 s, plus 0.5 s inter-scan gap, yielding an effective *TR* = 2.1 s; *TE* = 30 ms; matrix size: 64 × 64 × 28). Twenty-eight slices per volume were obtained with no inter-slice gap, providing full coverage of temporal and occipital lobes, but only partial coverage of the upper portion of the cerebrum. Specifically, coverage excluded superior portions of the somatosensory cortex, motor cortex and superior parietal lobe. A whole-brain high-resolution T1-weighted anatomical image was also obtained within-session prior to the first functional run using a 3D gradient-echo parallel acquisition sequence (MPRAGE; GRAPPA acceleration factor = 2; voxel size = 1 × 1 × 1 mm; *TR* = 2.3 s; *TE* = 2.98 ms; Flip angle = 9°; matrix size: 192 × 256 × 256).

### Univariate statistical analyses

Imaging data were analyzed using the AFNI software package (Cox, 1996). All functional scans were motion corrected using a 3D rigid body transform (AFNI *3dvolreg*) registered to the first functional volume of the first run. Statistical parametric maps were created using a general linear model (GLM, AFNI 3*dDeconvolve*) composed of six regressors; five condition regressors (Fall-Fast, Fall-Slow, Rise-Fast, Rise-Slow, Steady-State), and a single motion parameter estimate calculated as the root mean square of the six movement estimates derived from the motion correction step. Each task predictor was convolved with a canonical hemodynamic response function. Group statistical maps were created by registering each subject-wise map to a standard template (the TT_N27 “Colin” brain template) in the stereotaxic space of Talairach and Tournoux (1988), using an automatic registration procedure (the AFNI *@auto_tlrc* script, least-squares cost function). Each statistical map was then resampled to a resolution of 1 mm^3^ and spatially filtered with a 5 mm FWHM Gaussian kernel.

Statistical contrasts were performed via *t*-tests at the group level as follows: the Steady-State condition was contrasted with the four combined FM conditions to identify regions of greater sensitivity to time varying vs. static components of acoustic signals. The second and third contrasts identified voxels sensitive to either the rate or direction of FM sweeps (with Steady State condition set as a condition of no interest). For modulation rate, we contrasted (Rise-Fast + Fall-Fast) vs. (Rise-Slow + Fall-Slow); for sweep direction, we contrasted (Rise-Fast + Rise-Slow) vs. (Fall-Fast + Fall-Slow). Contrasts were thresholded at *p* < 0.05 corrected for multiple comparisons based on a voxel-wise threshold of *p* < 0.002 and a cluster size threshold of 971 mm^3^ (estimated using a 10,000-iteration Monte Carlo procedure, accounting for observed mean spatial blurring in each dimension; AFNI *3dClustSim*).

### Multivariate statistical analysis

**Figure 2:**
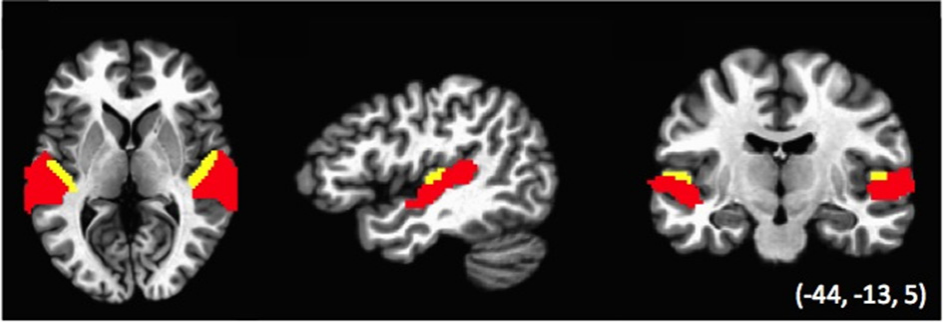
**ROIs used in the Relative similarity analysis (RSA)**. Areas in red (Auditory cortex ROI) are auditory-functionally defined regions of left and right temporal lobe; areas in yellow (Heschl's gyrus ROI) correspond to the anatomically defined Heschl's gyrus in a standard atlas (see Linke et al., 2011). Note that the two sets of ROIs do not overlap.

Data were also analyzed using representational similarity analysis (RSA; Kriegeskorte et al., 2008), to examine the relative similarity of the voxel activation pattern across conditions. Analyses were performed within two regions of interest (ROIs): auditory cortex defined functionally across the temporal lobe, and Heschl's gyrus, with both ROIs based on regions defined within a previous study (Linke et al., 2011; see Figure 2). The Auditory cortex ROI was defined as regions of temporal cortex that was activated in the Linke et al. study during the encoding, maintenance and comparison of tone stimuli. This ROI subtended the anterior and posterior plane of STG and STS. The Heschl's gyrus ROI was identified anatomically using a standard atlas. Note that the two ROIs were non-overlapping such that the Auditory ROI excluded voxels falling within the Heschl's ROI and vice-versa. We performed RSA separately for activation patterns within the ROIs of the left, right and combined hemispheres, for a total of six analyses.

Voxel activation patterns were computed for each subject on each of the five stimulus types using a GLM as specified above, but with no spatial smoothing, and with separate GLM maps obtained for even and odd runs. This yielded two sets of five statistical maps per subject. Beta coefficients for each statistical map were ROI masked and subjected to Spearman correlations between even and odd runs for each combination of conditions, yielding a 5 × 5 similarity matrix for each subject. Next, statistical contrasts were performed groupwise to investigate the dissimilarity of the two dynamic features of interest. RSA for direction of modulation was assessed by performing a pairwise *t*-test for coefficients in the rising vs. falling conditions, collapsing across the two rate conditions; RSA for rate was assessed by performing a pairwise *t*-test for the fast and slow conditions, collapsing across the two direction directions. Significant differences in an ROI indicated this region differentially encodes information regarding the categories of stimuli under investigation.

## Results

### Univariate analysis

**Figure 3:**
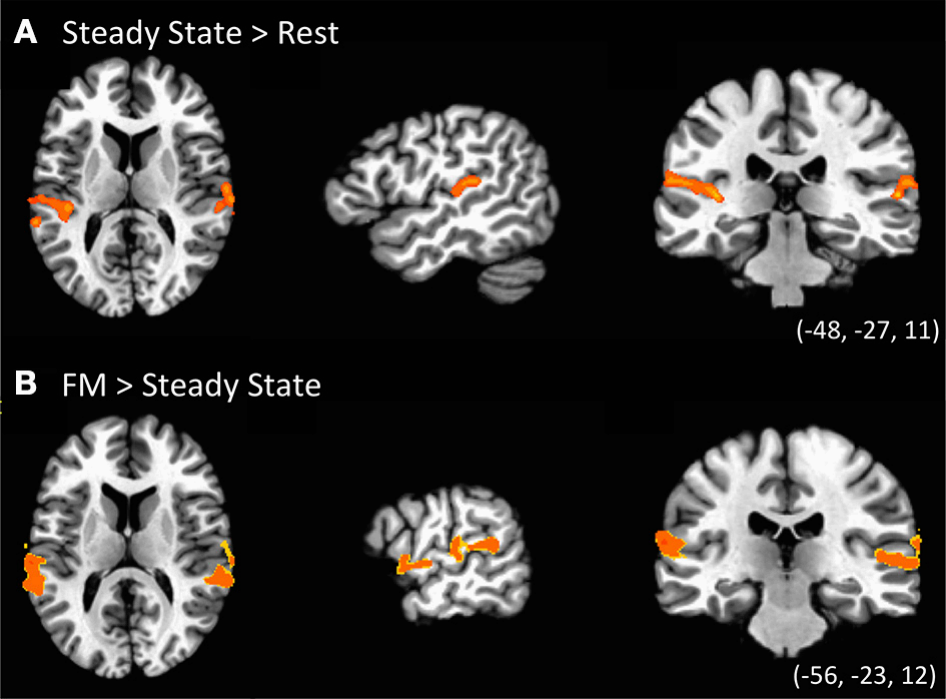
**Univariate statistical contrasts. (A)** Steady state condition vs. rest reveals bilateral activation in superior temporal gyrus within and around primary auditory cortex. **(B)** Frequency-modulated (FM) sweeps vs. steady state, showing greater activation for FM sounds in the superior temporal plane of both right and left hemispheres. Group statistical maps are superimposed on a standardized template. Voxel-wise threshold: *p* < 0.002, corrected to *p* < 0.05 for a 971 mm^3^ cluster extent.

The first contrast investigated the existence of specialized regions for processing FM sounds compared to spectrally similar steady-state sounds, and was computed using a one-sample *t*-test for the Steady-State predictor vs. zero (Figure 3A, Table 2). Results revealed clusters of activation in bilateral posterior STG in and around Heschl's gyrus. We next contrasted the combined FM conditions vs. the Steady State condition. As indicated in Figure 3B and the lower portion of Table 2, we found clusters of activation throughout bilateral auditory cortex peaking in portions of superior temporal gyrus (STG) both anterior and posterior to Heschl's gyrus, and extending more ventrally toward superior temporal sulcus (STS). This effect was more pronounced in the left than right hemisphere, taking into account the total size of the two separate clusters in L-STG/STS. A significant cluster was also observed in the right supramarginal gyrus (SMG).

**Table 2:** **Location and size of the peak voxel activation for the univariate analysis**.

**Region**	**Talairach Coordinates**			**Size (*mm*^3^)**
	***X***	***Y***	***Z***	
**STEADY vs. REST**				
L STG	−43	34	14	3667
R STG	56	28	8	1404
**DYNAMIC > STEADY**				
L STG	−61	24	13	2166
L STG	−57	8	3	1514
R STG	56	8	3	3328
R SMG	44	58	34	1360

*Corrected α = 0.05; voxel-wise threshold: p < 0.002; cluster size threshold = 971 mm^3^*.

We also sought to identify regions responsible for processing either the direction or rate of FM sweeps. For the effect of rate (fast vs. slow), the two levels of direction were conflated: (Rise-Fast + Fall-Fast) vs. (Rise-Slow + Fall-Slow). In a similar fashion, the effect of direction was examined by collapsing over rates: (Rise-Fast + Rise-Slow) vs. (Fall-Fast + Fall-Slow). Neither of these contrasts yielded significant difference in either direction at a threshold of significance corrected for multiple comparisons. This lack of effect persisted even when not controlling for cluster extent at this same voxel-wise significance threshold.

### Multivariate analysis

**Figure 4:**
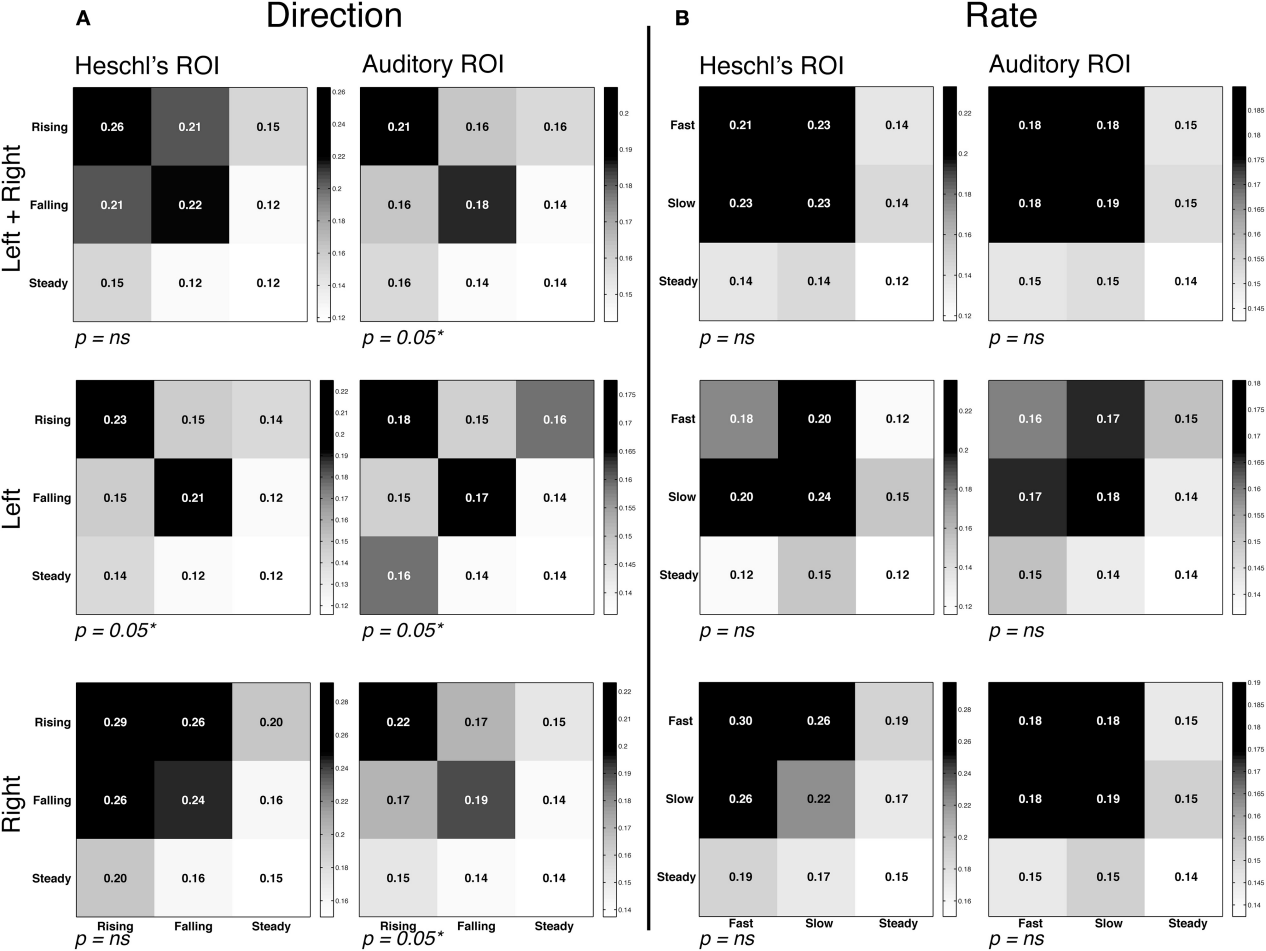
**Representational similarity analysis**. Mean correlation of activity patterns between even runs (rows) and odd runs (columns), at each region of interest (see Figure 2). Within-category correlations correspond to the diagonal of the matrix; between-category correlations correspond to off-diagonal values. Higher similarity is depicted by higher correlations and darker shading. Note that overall correlation ranges varied by ROI, and therefore different intensity scales are used in each ROI in order to highlight differences in the relative degree of similarity. Also note that statistical analyses compared the correlation coefficients of cells, and not the significance of each correlation on its own. Thus, *P*-values correspond to the comparison of between vs. within-condition correlation coefficients for the rapid temporal conditions (the upper left 2 × 2 square of each matrix). In all graphs the third row/column corresponds to the steady state condition. **(A)** Correlation matrices for the direction contrast, collapsing over the rate manipulation. We observed significantly greater representational similarity within the FM conditions (i.e., rising-rising and falling-falling) compared to between conditions (rising-falling/falling-rising). **(B)** Correlation matrices for the rate contrast, collapsing over the direction manipulation. No effect of rate was observed, marked by similar degrees of similarity within condition (fast-fast/slow-slow) and between condition (fast-slow/slow-fast).

The RSA analysis measured the similarity of voxel activation patterns for FM rate and direction within a given ROI. RSA matrices and contrasts are visualized in Figure 4. Within each matrix in Figure 4, the correlations between each grouping of conditions is plotted, with the relative intensity of each square denoting the degree of similarity; statistical analyses then contrasted the correlation coefficients in order to assess whether representational similarity within each ROI was different for the conditions of interest. The first contrast examined whether different directions of FM (rising vs. falling) yielded different patterns of activation. The results revealed strong evidence of direction-specific activity patterns in left and right Auditory ROIs, and in the left Heschl's gyrus ROI. This is best visualized by stronger correlations within each sub-plot for FM conditions along the diagonal (rising vs. rising and falling vs. falling) compared to the off-diagonal (rising vs. falling). In contrast, the RSA analysis did not reveal strong evidence for differentiation within the rate manipulation, marked by a failure to find significantly greater similarity of activation patterns within-category vs. between-category. These results suggest that auditory cortex is generally more sensitive to changes in direction than to changes in the rate of frequency-modulated sweeps for the rapid FM acoustic features explored in this experiment.

## Discussion

Spoken language comprises a dynamic and broadband acoustic signal made up of many types of temporal and spectral features. In the present study we were interested in one specific aspect of speech, the rapid temporal frequency modulations that are used to signal phonetic contrasts such as place of articulation. Our stimuli involved non-speech sounds that isolated specific characteristics of frequency modulation (FM) namely direction and rate of frequency changes.

We first investigated whether FM sweeps and steady-state sounds elicited different responses in large-scale brain activity patterns. By contrasting brain regions that were activated in response to the two classes of stimuli (FM sweeps vs. steady-state sounds), we were able to demonstrate that there are indeed differences in both the extent and magnitude of activation within both core and belt auditory cortex. This finding is consistent with prior studies showing that auditory cortex is generally organized in a way that codes for increasing complexity of auditory information as it extends outward from primary auditory cortex to regions that surround it (e.g., Rauschecker et al., 1995; Hall et al., 2002; Chevillet et al., 2011). The present study fits well with such findings, illustrating that this effect can be driven by rapid FM characteristics of sounds. The steady-state sounds used in the current study were as spectrally complex as the FM sweeps; the only difference was the time varying nature of the FM sounds.

We also observed an interesting pattern of lateralization of activation in response to frequency-modulated sweeps, such that the left hemisphere displayed a greater extent of activation than the right hemisphere. This finding lends further support to the role of FM in language processing given the theory that the left hemisphere is specialized for processing the salient auditory features that are the components of more complex acoustic signals such as speech. The finding is in line with previous research that demonstrated that temporal modulation of auditory inputs yield stronger left hemisphere activation compared to steady-state stimuli (e.g., Zatorre and Belin, 2001; Hall et al., 2002) and that congruent effects occur for speech stimuli incorporating these rapid temporal characteristics (Joanisse and Gati, 2003). This suggests that sensitivity to rapid temporal cues reflects a fundamental specialization of left auditory cortex for processing time varying acoustic signals, both for speech and non-speech.

One note about this interpretation is in order however. We also observed a somewhat greater extent of left-hemisphere activation for the steady-state condition alone even though no FM cues were present in that case (Figure 3A). The explanation for this appears to be the periodic nature of the IRN stimulus itself. Although the perceived pitch of the IRN stimulus was held constant in the case of the steady-state condition, it nevertheless contains a degree of amplitude modulation (visible as dark vertical bands in Figure 1A, center), and this is itself a rapid temporal feature. Similarly, the brief nature of the auditory stimuli (250 ms) yield a rapid rise and fall in amplitude envelope during stimulus presentation. We suggest that either of these amplitude modulation characteristics would tend to drive greater response in left vs. right auditory cortex due to their rapid temporal nature. Notwithstanding, this bias cannot fully explain the greater extent of left hemisphere activation in the second univariate analysis, where FM sweeps were contrasted with steady state stimuli. Here again, the left-hemisphere preference persisted even though amplitude modulation features were held constant between the steady-state and FM stimuli.

We next examined whether either the rate or direction of FM sweeps elicited differences in the extent and/or magnitude of activation within auditory cortex. Two contrasts were performed, for the rate and direction of frequency modulation. Neither yielded significant differences, even when a more lenient statistical approach was adopted that allowed for smaller extents of significant voxels. The null findings are not surprising considering what is currently known about the auditory cortex as it pertains to processing FM, the response properties of neurons located in this region, as well as their organization on a macroscopic level. For instance, using a similar univariate approach, Hsieh et al. (2012) also failed to observe macroscopic regions that differentiated either the direction or rate of FM sweeps. And although electrophysiological work in animals (Mendelson et al., 1993; Tian and Rauschecker, 2004) has revealed the existence of rate-selective and direction-selective neurons for rapid temporal FM stimuli, the selectivity of these neurons is not strictly exclusive. Though some neurons appear to respond more strongly to a specific rate or direction, they also fire at lower levels for other stimulus types as well. Moreover, such neurons are not distributed in a topographically consistent manner, such that a neuron sensitive to one direction or rate might be located immediately adjacent to a neuron sensitive to different parameters. The coarse resolution of fMRI means it would be rather difficult to capture such effects using traditional univariate approaches.

### Multivariate analysis identifies FM-sensitive regions

To address this, RSA was used to perform MVPA analyses in left and right hemisphere auditory cortex. This analysis approach is especially adept at detecting differences in stimulus-dependent patterns of brain activity in the absence of differences in either the magnitude or location of activation. We first investigated whether the direction of frequency-modulated IRN sweeps elicited differentiable patterns of brain activity. We found significant dissimilarity in the patterns of activation for rising vs. falling sweeps bilaterally. This effect was strongest for the broader Auditory cortex ROI, compared to the more narrowly proscribed Heschl's gyrus, suggesting that portions of the belt region outside primary auditory cortex are tuned to FM features of sounds. Indeed, the cytoarchitectonic organization of neurons within core and belt regions of auditory cortex varies considerably and this has implications for the types of analyses that will prove useful in identifying differences in brain activations in response to different acoustic stimuli. While neurons within the auditory core are comprised of smaller, more densely packed neurons, the belt regions that surround it consist of larger and less densely packed neurons (Sweet et al., 2005). These neuroanatomical divisions might serve to drive differences in the representational capacity of these different regions for certain types of acoustic features.

One caveat is in order here: the direction of modulation was manipulated by modifying the initial frequency of the tone sweep. As a result the falling stimulus necessarily had a higher initial frequency than the rising stimulus. Because of this, it is possible that differences between rising and falling stimuli were due to these spectral differences, rather than their FM characteristics. Note that this confound represented what we felt was the least problematic of different possible ways to manipulate direction of frequency modulation; the alternative would have been to create sweeps that involve frequency modulations with different initial and final frequencies such that the overall frequency range of the sweeps was identical but in opposing directions. However, this would have required having a different final steady-state frequency component for rising and falling stimuli (cf. Table 1), which because of its duration would have yielded a much stronger spectral confound than what was found here. We do note that our findings are convergent with what Hsieh et al. (2012) found for direction-sensitivity however; their study also manipulated FM direction but controlled for the overall frequency range of both stimulus types by using different stimulus durations. The fact that both our studies have identified direction-sensitive patterns of activation in auditory cortex supports the interpretation that these effects are due to temporal, and not spectral, characteristics of the stimuli.

### Failure to identify effects of FM rate at this time scale

Notably, we failed to find similar evidence of sensitivity to the FM rate manipulation in our experiment. This is surprising given a prior affirmative finding by Hsieh et al. (2012) for slower-rate FM sweeps. We argue that the reason for this discrepancy is the short, rapid FM sweep contrasts used in the present study. A study by Schwab et al. (1981) seems especially relevant in this regard. The authors examined adult English speakers' sensitivity to the duration, rate and extent (i.e., frequency range) of a formant transition cue, in the context of discriminating the syllables /ba/ and /wa/. The acoustic characteristics of these formant transitions were very similar to the non-speech FM stimuli used in the present study. The authors found that listeners were sensitive to both the duration and the extent of a formant transition cue; however the rate of frequency change alone was not sufficient for discriminating among phoneme categories. Listeners also appeared to label the two stimuli based on a criterion that weighted both extent and duration equally, such that if the frequency extent (Hz) times the duration (ms) exceeded 23,000, it would be labeled as a glide (/w/), otherwise it would be labeled as a stop (/b/). Note that if we use the same metric for our stimuli, both the “fast” and “slow” rates would fall on the high side of this criterion, due to the relatively narrow frequency extent that we used here (600–1200 Hz).

Overall then, the null result for rate could be interpreted as showing that the phonetic labeling criterion identified by Schwab et al. is in fact recapitulated by the cortical organization within the auditory system. Ultimately however, it would be important in future work to manipulate the extent and duration of FM information in a way that better captures the use of those parameters in phoneme contrasts.

The fact that Hsieh et al. (2012) did find an effect of FM rate appears to also be due to the acoustic parameters that were being used in that study. As noted above, their FM rate manipulation was on a generally slower order than what was used here, and was more in keeping with tonal contrasts observed in some languages. Thus, we are not claiming that modulation rate is never important for speech perception, or that auditory cortex is generally insensitive to such cues. Rather we argue that this is a relatively weak cue at the rapid time scale being considered in this study. Put another way, temporal cues are argued to be relevant to speech cues at multiple grain sizes including phonemes, tones, word-level stress and sentence level stress (Giraud and Poeppel, 2012; Henry and Obleser, 2013). It appears, however, that the types of temporal cues used for different levels of processing may be distinct, and indeed governed by somewhat different principles of neural processing (Obleser et al., 2012).

That said, there is an alternative possibility for our failure to find an effect of sweep rate, which is that our methodology was not sufficiently sensitive to observe such a difference. We used a rapid, jittered, event-related fMRI paradigm that optimized the ability to present single trials in random order. Our motivation to adopt this design over a block design was that this second option involves repetitive presentations of a given stimulus category within each block, which can inadvertently direct subjects' attention toward the feature of interest for that block. This in turn could yield undesirable effects given our goal of measuring basic perception of acoustic features in auditory cortex. Additionally, the periodic presentation of stimulus trains within each block is itself a temporal feature (i.e., the rise and fall of an amplitude envelope), and this might also drive auditory temporal processes that are separate from the single-stimulus properties that were of interest in our study. Thus, we felt an event-related paradigm, especially one that presented stimuli at irregular intervals, would yield the clearest picture with respect to basic auditory cortical sensitivity to frequency modulation.

On the other hand, it is well recognized that block designs generally yield better statistical power than event-related designs by maximizing the contrast of task-driven BOLD response against background noise. It is therefore conceivable that we would have found effects of sweep rate had we adopted a block design. We do note however that there was sufficient power in our experiment to find effects of sweep direction using the same analyses. Given that the same number of trials was employed for both manipulations, we can at the very least conclude that the effect of rapid FM direction is appreciably stronger than that of FM rate.

### Implications for phonetic processing

Recognizing speech extends beyond just recognizing the component acoustic features of the speech stream. What we have examined here is an early step in a processing chain that involves matching the acoustic features to phonetic categories and/or articulatory gestures, and proceeding onwards to lexical, semantic and syntactic analyses (Hickok and Poeppel, 2004; Rauschecker and Scott, 2009). So for example, other studies have found that phonetic perception, in which speech sounds are categorized or discriminated, specifically engages STS areas that are ventral to the STG regions of interest in this study (Liebenthal et al., 2005; Joanisse et al., 2007). Likewise, sounds that are perceived as speech yield fMRI effects that are differentiable from those observed for acoustically similar non-speech sounds, again supporting the view that the phonetic content of speech engages selective brain mechanisms beyond simple acoustic feature detection (Vouloumanos et al., 2001; Liebenthal et al., 2005). So in short, what we have identified in the present study might be best thought of as the acoustic precursors to acoustic-phonetic perception, and cannot explain the entire process of phoneme recognition during speech perception. We do predict however that speech sounds that contain similar acoustic cues to the non-speech cues manipulated here will also yield similar effects in the regions of auditory cortex, supporting the view that at an early point in processing there is no strong distinction between how speech and non-speech sounds are processed.

## Conclusions

We used fMRI to examine the organization of human auditory cortex for processing frequency modulated sounds. The results yield insights into how auditory cortex processes acoustic elements that are fundamental to phoneme perception. Using IRN stimuli that approximate both spectral and rapid temporal speech characteristics, we observed that FM sweeps activated a broader set of regions of auditory cortex compared to control sounds that were spectrally similar but not frequency-modulated. More importantly, multivariate analyses demonstrated the existence of direction-specific activity patterns at a microscopic level in both left and right auditory cortex. The findings add to a growing literature supporting the view that auditory cortex contains neural populations specifically tuned to detecting at least some types of acoustic features important for phonetic processing. Moreover it illustrates the utility of applying multivariate data analysis techniques such as RSA to elucidate differences in patterns of brain activity when gross regions of activation overlap.

### Conflict of interest statement

The authors declare that the research was conducted in the absence of any commercial or financial relationships that could be construed as a potential conflict of interest.
